# Structure of the *Plasmodium*-interspersed repeat proteins of the malaria parasite

**DOI:** 10.1073/pnas.2016775117

**Published:** 2020-11-30

**Authors:** Thomas E. Harrison, Adam J. Reid, Deirdre Cunningham, Jean Langhorne, Matthew K. Higgins

**Affiliations:** ^a^Department of Biochemistry, University of Oxford, Oxford OX1 3QU, United Kingdom;; ^b^Parasite Genomics, Wellcome Sanger Institute, Cambridge CB10 1SA, United Kingdom;; ^c^The Francis Crick Institute, London NW1 1AT, United Kingdom

## Abstract

The deadly symptoms of malaria occur as *Plasmodium* parasites replicate within blood cells. Members of several variant surface protein families are expressed on infected blood cell surfaces. Of these, the largest and most ubiquitous are the *Plasmodium*-interspersed repeat (PIR) proteins, with more than 1,000 variants in some genomes. Their functions are mysterious, but differential *pir* gene expression associates with acute or chronic infection in a mouse malaria model. The membership of the PIR superfamily, and whether the family includes *Plasmodium falciparum* variant surface proteins, such as RIFINs and STEVORs, is controversial. Here we reveal the structure of the extracellular domain of a PIR from *Plasmodium chabaudi*. We use structure-guided sequence analysis and molecular modeling to show that this fold is found across PIR proteins from mouse- and human-infective malaria parasites. Moreover, we show that RIFINs and STEVORs are not PIRs. This study provides a structure-guided definition of the PIRs and a molecular framework to understand their evolution.

## Significance

The *Plasmodium* parasites that cause malaria replicate within blood cells of an infected host. These parasites send a small number of proteins to infected blood cell surfaces, allowing them to bind host molecules but also risking their detection by the host immune system. These proteins have diversified into large families, allowing the parasite to avoid detection by using antigenic variation. The most ubiquitous of these families is the *Plasmodium*-interspersed repeat (PIR) protein family. Here we present the structure of a PIR protein, revealing the architecture of its ectodomain and showing how it has diversified. Finally, we use structure-guided methods to understand which small variant surface antigen families are PIRs and to understand their evolution across malaria parasites.

The symptoms of malaria occur as *Plasmodium* parasites replicate within blood. This is a rich environment, replete with the nutrients required for growth and providing the opportunity for transmission by blood-sucking insects. However, blood also contains much of the machinery of the host immune defense. To survive under immune attack, *Plasmodium* parasites have evolved to replicate while hidden within host cells. Only a few parasite proteins are exposed on host cell surfaces, and these have mostly diversified into large protein families, allowing a population survival strategy based on antigenic variation (1, 2).

The best understood of the infected-erythrocyte surface protein families is the PfEMP1, members of which interact with human endothelial receptors, causing infected erythrocytes to adhere within the vasculature away from splenic clearance (1, 3, 4). However, PfEMP1 are found only in *Plasmodium falciparum* and the closely related *Laverania*. More ubiquitous across *Plasmodium* species are families of small variant surface antigens (VSAs) (1). These include the CIRs of *Plasmodium chabaudi* (5) and the VIRs of *Plasmodium vivax* (6), often known as the “*Plasmodium*-interspersed repeats” (PIRs) (7). The PIRs can be very abundant, with thousands of members in some genomes (8). However, whether some families of small VSAs in the *Laverania* and more distantly related *Plasmodium* species are part of the PIR superfamily is unclear. For example, the RIFINs and STEVORs of *P. falciparum* (9–11) were proposed to be PIRs due to their sizes, cellular locations, and the presence of shared sequence elements within intron regions of their genes (12). However, differences in gene structure and a low protein sequence identity make this assignment uncertain (7). Are the small VSAs part of a larger superfamily with related functions, or are they different protein families with different roles?

Also uncertain is whether the small VSAs are universally found on infected erythrocyte surfaces. Studies have located PIRs on or close to the surfaces of blood cells infected with *P. vivax* (6), *Plasmodium yoelii* (13), and *Plasmodium berghei* (14). Similarly, RIFINs and STEVORs have been located to surfaces of *P. falciparum*-infected erythrocytes (10, 15–17). Indeed, natural infection with *P. vivax* induces antibodies that target VIRs (18), while unusual RIFIN-targeting antibodies found in adults in malaria endemic regions of Africa also recognize infected erythrocytes (19–21). These studies suggest that the small VSAs are molecules of infected blood cell surfaces and indicate that family expansion and diversification have occurred to allow them to undergo antigenic variation. However, other studies have cast doubt on the universality of this model, indicating an intracellular location for some small VSAs (22–24) or showing them be expressed in other life cycle stages of the parasite, including merozoites or gametocytes (23, 25–27). Has diversification of these small VSA families led to their use at different life cycle stages and different cellular locations during infection?

A number of recent studies indicate that small VSAs have important functions. First, *P. chabaudi* introduced into mice through mosquito bite are less virulent than those introduced by direct injection of infected blood (28). The major differences in gene expression in these parasites are in the *cir* gene repertoires, with a broader range of *cir* genes expressed on mosquito transmission (28). Different *cir* genes are also transcribed during the chronic phase of a mosquito-transmitted *P. chabaudi* infection compared with those expressed during the acute phase (29). When passaged in naïve mice, these chronic parasites are more virulent than those from acute stages of infection. Indeed, a more virulent *P. chabaudi* strain, PcCB, expresses more of the *pir* genes associated with chronic infection than a less virulent strain, PcAS (30). These findings combine to suggest an as-yet unknown role for PIRs in modulating the virulence of infection. One possible mechanism in some *Plasmodium* species might be through causing adhesion of infected cells within the vasculature, allowing avoidance of splenic clearance. Indeed, VIRs from *P. vivax* are proposed to cause infected-reticulocytes to adhere to endothelial receptors including ICAM-1 (22, 31), while RIFINs and STEVORs can cause infected erythrocytes to adhere to uninfected erythrocytes, by interacting with blood group antigens or glycophorin C (16, 32). Alternatively, subgroups of RIFINs have been shown to interact with human inhibitory immune receptors, such as LILRB1 and LAIR1 (17). The LILRB1-binding RIFINs mimic the natural ligand of LILRB1, MHC class I, allowing the RIFIN to inhibit markers of natural killer cell activation, most likely reducing parasite clearance (33). These studies imply wide-ranging roles for the small VSAs in both mammalian and insect hosts.

The important roles emerging for different small VSAs highlights the need to understand these mysterious parasite protein families in greater detail. In particular, are they members of the same *Plasmodium* superfamily, evolved to perform similar functions during infection, or do the small VSAs represent different protein families with different roles? To explore this question, we determined the structure of the extracellular domain of a CIR protein from *P. chabaudi*. We compared this structure with that of the variable domain of a LILRB1-binding RIFIN (33) and used structure-guided sequence analysis to predict which small VSAs are part of the PIR superfamily. This provides a framework for understanding PIR protein function and evolution.

## Results

### The Structure of a *P. chabaudi* PIR Protein Ectodomain.

**Fig. 1.**
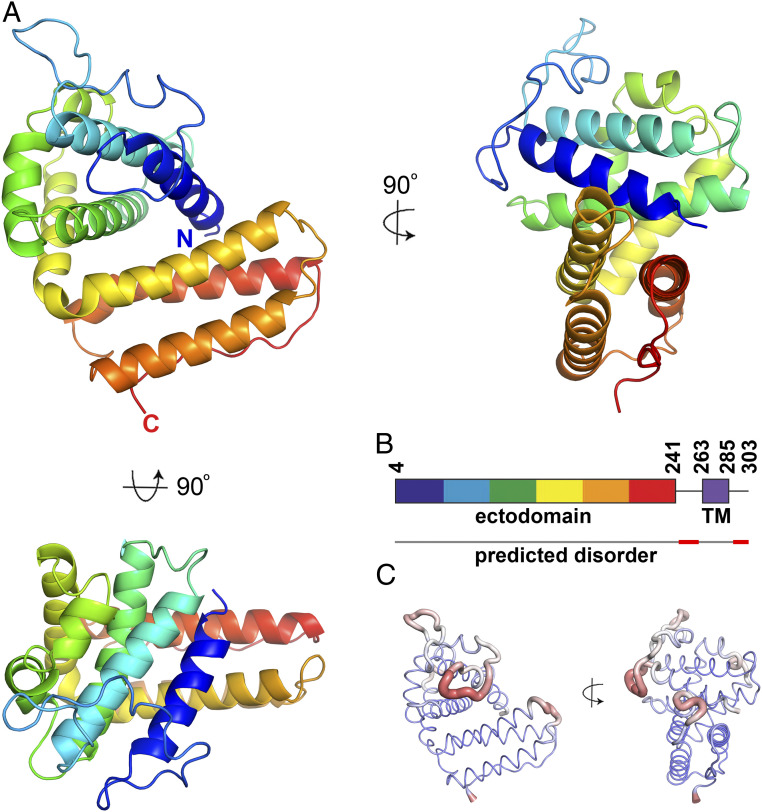
The structure of a *P. chabaudi* PIR protein. (*A*) The structure of the CIR protein ectodomain from PCHAS_1200500 is shown in rainbow representation, with the N terminus in blue and the C terminus in red. (*B*) A schematic of the full CIR protein PCHAS_1200500 showing the ectodomain (rainbow) and the transmembrane helix (TM; purple). Below the schematic is a line representing the predicted disorder across the domain, with disordered regions in red. (*C*) The structure of the CIR domain represented as a wire, with the thickness scaled according to the crystallographic B factor of this region of the model. Broad, red regions of wire indicate more disordered regions of the protein domain.

To determine the structure of a member of the PIR family, we focused on proteins from *P. chabaudi,* often known as CIRs. These consist of an N-terminal extracellular domain ranging in size from 28 kDa (236 residues) to 133 kDa (1,331 residues) in the AS strain. This is followed by a predicted transmembrane helix and small intracellular peptide. We assessed the expression of a panel of seven of the smaller CIR ectodomains (*SI Appendix*, Fig. S1). Five of these were expressed in HEK293F cells and were subjected to crystallization trials. Crystals formed for PCHAS_1200500 and contained three copies in the asymmetric unit. A complete dataset was collected to 2.15 Å, and the structure was determined by Sulfur-SAD phasing, using anomalous scattering from the sulfur atoms found in the five disulfide bonds and six methionine residues in each monomer (Fig. 1*A* and *SI Appendix*, Table S1). A complete model was built for residues 5 to 242. Another 20 residues, which connect the ectodomain to the predicted transmembrane helix, are missing from the C terminus, suggesting that this domain is connected to the membrane through a flexible, disordered linker. Indeed, residues 242 to 258 are predicted to be disordered (Fig. 1*B*).

The CIR ectodomain adopts a compact α-helical structure, arranged into two distinct clusters of helices (Fig. 1*A*). It does not adopt the same fold as known *Plasmodium* surface protein modules, such as the DBL and CIDR domains found in the PfEMP1 proteins, and the DALI server did not identify a similar protein of known structure. The N-terminal half of the ectodomain is formed from five tightly packed helices connected by long loops largely lacking in secondary structure. This packs against the C-terminal portion of the ectodomain, which is folded as a cluster of three parallel helices, connected by short loops. The C-terminal end of the fold consists of a long stretch of residues lacking in secondary structure, running alongside the final two helices. Five disulfide bonds stabilize the fold, supporting the hypothesis that the CIRs are surface proteins, because disulfide bonds are characteristic of proteins located in the oxidizing extracellular environment. Two of the disulfide bonds lie within the loop between the first and second helices, and a third bond stabilizes the loop between helices 2 and 3. The final two disulfide bonds link helices within the core of the domain, with one linking helices 1 and 6 and another linking helices 7 and 8.

The CIR ectodomain is predicted to be tethered to membranes through a C-terminal transmembrane helix. The surface opposite this attachment site is dominated by a long loop that links helices 1 and 2. This is one of the most flexible regions of the domain, containing the residues with the highest crystallographic B factors (Fig. 1*C*). In addition, two copies of the domain in the asymmetric unit of the crystal have missing density in this region, with chain B missing residues 45 to 48 and chain C missing residues 25 to 29, again indicating flexibility. Other regions of higher B factors include the loops linking helices 4 and 5 and helices 6 and 7, both lying on the same side of the domain. For any CIR proteins found on infected erythrocytes, this surface will be the most exposed to the immune system. With disorder reducing the likelihood of antibody attachment, this suggests that flexible and disordered sequences have evolved on this exposed surface to decrease the likelihood of immune detection.

### Diversity and Conservation within the CIR Proteins.

**Fig. 2.**
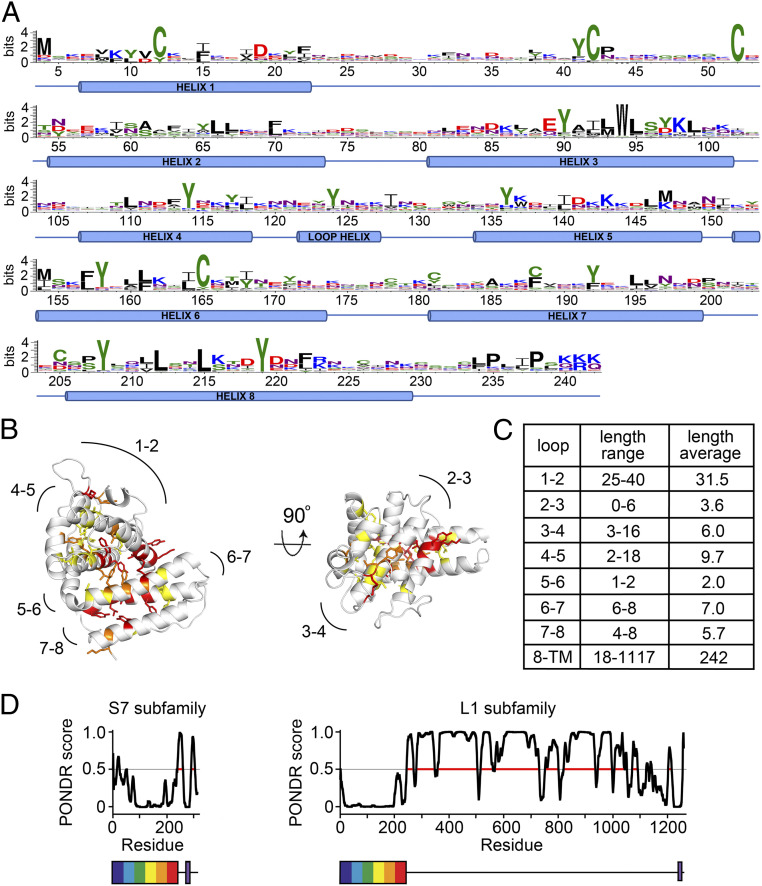
Conservation and diversity in the CIR proteins. (*A*) All CIR sequences from the AS strain of *P. chabaudi* were aligned against the structure of PCHAS_1200500, and a sequence logo was generated, numbered according to PCHAS_1200500. (*B*) Residues most chemically conserved across the CIR proteins of the AS strain are shown as sticks. Residues are color-coded by Shannon sequence entropy: 0.75 to 1.0 in yellow, 0.5 to 0.75 in orange, and <0.5 in red. Lower sequence entropy indicates greater conservation of side chain chemical properties. (*C*) A table of the lengths of the loops and of the linker between the PIR domain fold and the transmembrane helix. (*D*) Prediction of disorder (PONDR score) for members of the S7 and L1 CIR protein subfamilies, in each case representing the protein closest in sequence to the sequence logo for that protein family, PCHAS_0500200 for the S7 family and PCHAS_0601000 for the L1 family. Prediction of disorder, determined using PONDR, is plotted against residue number. Below the plots are representations of the two proteins, showing the PIR protein domain as a rainbow box and the transmembrane helix in purple.

To understand the degree to which CIR proteins diversify, we analyzed all 198 CIR sequences from the AS strain of *P. chabaudi* by aligning them to the structure of PCHAS_1200500. A conservation LOGO for sequences aligned in this way identified the majority of conserved residues as cysteines or aromatic residues, found predominantly on helices (Fig. 2*A*). The Shannon sequence entropy, which takes into account the chemical properties of amino acid side chains in assessing chemical conservation, was calculated for each residue, and these scores were plotted on to the PCHAS_1200500 structure (Fig. 2*B*). The majority of chemically well-conserved residues, with low entropy, were found in the core of the protein, stabilizing the packing of the helices and indicating conservation of the protein fold. Two of the disulfide bonds, corresponding to Cys12-Cys165 and Cys42-Cys52, were also well conserved. In contrast, the surface of the protein shows no conservation, as would be expected for a family of antigenically distinct proteins under immune pressure. This lack of conservation is particularly stark in both the sequence and the length of the surface loops that link the helices (Fig. 2). This is reminiscent of the DBL and CIDR domains of *Plasmodium* parasites, which also have conserved core aromatics and disulfide bonds to stabilize their fold, while showing great diversification on the surface (34–37).

The core PIR protein domain starts within five residues of the N terminus of each of these 198 CIR proteins. However, there are significant differences in the length and nature of the linker between this domain and the transmembrane helix, which ranges from 18 residues to 1,117 residues. The CIRs have previously been divided into the S family and the L family, with the length of the linker the primary difference between these groups (Fig. 2*D* and *SI Appendix*, Fig. S2). For example, in the S7 CIR subfamily, which contains PCHAS_1200500 and is associated with acute infection (29), this linker ranges from 20 to 68 residues, with an average of just 37. In contrast, in the L1 family, whose expression is associated with chronic infection, it is typically much longer, ranging from 59 to 1117 residues, with an average of 533. Analysis using five different predictors of protein disorder suggests that this portion of the ectodomain is largely disordered in all CIR protein classes (Fig. 2*D* and *SI Appendix*, Fig. S2). This indicates that the ectodomains of all CIRs share a similar architecture, with a structurally conserved yet sequence-diverse N-terminal fold connected to a transmembrane helix through a flexible linker of highly variable length.

### Which Other Plasmodium Protein Families Share the PIR Protein Fold?

**Fig. 3.**
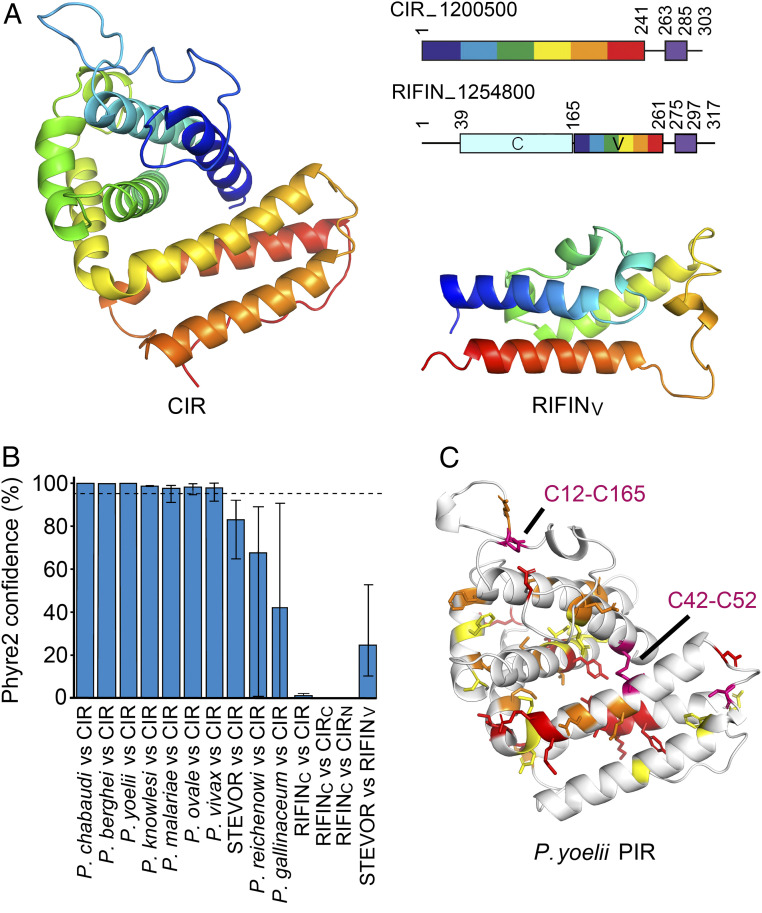
Structure-guided determination of membership of the PIR protein superfamily. (*A*) Comparison of the structure of the CIR protein ectodomain with that of the variable domain of the RIFIN (RIFIN_V_). Both are shown as rainbow representation from N terminus (blue) to C terminus (red). The top right panel shows schematics for these two proteins, with numbers referring to the position in the protein sequence. Both have a transmembrane helix, represented as a purple box, close to the C terminus. The RIFIN has a variable domain (V; rainbow representation) and a constant domain (C; pale blue). (*B*) The Phyre2 confidence scores for modeling of small VSAs from different *Plasmodium* species using the known structures of the CIR protein, its C-terminal lobe (CIR_C_: residues 152 to 241), its N-terminal lobe (CIR_N_: residues 4 to 151), or against the RIFIN_v_ domain. The dashed line represents the high confidence cutoff limit of 95%, and the error bars show the range of scores obtained from the 10 examples of each PIR protein modeled. (*C*) A model of a *P. yoelii* PIR protein extracellular domain, PY17X_094500, based on the CIR structure. Residues with a property entropy of 0.75 to 1.0 are in yellow, those with 0.5 to 0.75 are in orange, and those with <0.5 are in red. Two disulfide bonds shared with the CIR protein structure are shown in purple, with the relevant cysteine residues labeled.

We next asked whether the CIR protein fold is conserved across the small VSAs, using structural insight to define the PIR superfamily. Comparisons of the CIR structure with our structure of the variable domain of a RIFIN (PF3D7_1254800) (33) revealed very different architectures (Fig. 3*A*). The RIFINs are mostly small proteins of 30 to 50 kDa with an N-terminal semiconserved domain and a C-terminal variable domain, followed by a putative transmembrane helix (32, 38). A hydrophobic region lying between the variable and constant domains has been suggested to act as a second transmembrane helix, but recent studies show that it is more likely part of the ectodomain (32, 38, 39). The RIFIN variable domain consists of a three α-helix core decorated with complex loops, which include the LILRB1-binding site. This structure most closely resembles the three C-terminal helices of the CIR protein; however, the topology and arrangement of these helices do not match. It is also unlikely that the N-terminal conserved domain of the RIFIN is structurally similar to the CIR protein structure, owing to the size difference. While the CIR protein domain of PCHAS_1200500 is 241 residues long, the N-terminal domain of RIFIN 1254800 is just 124 residues long. Therefore, the CIR ectodomain fold is too large to be accommodated within the RIFIN conserved domain. These findings suggest that RIFINs and CIRs are not structurally equivalent but rather are two distinct classes of *Plasmodium* surface proteins.

We next aimed to determine whether other small VSAs, which have not yet been structurally characterized, adopt the same fold as either a CIR or a RIFIN. We studied diverse small VSA sequences from the rodent-infective *Vinckeia* clade, the monkey- and human-infecting *Plasmodium* clade, ape-infecting *Laverania* species, and rodent- and bird-infective *Plasmodium* species, aiming to survey across the *Plasmodium* evolutionary tree (*SI Appendix*, Fig. S3). For each protein family, we selected 10 sequences, chosen to represent a range of sequence diversity (*SI Appendix*, Fig. S3). These were analyzed by structure-based threading using Phyre2 (40), generating homology models and providing a score that represents confidence in the model generated (Fig. 3*B* and *SI Appendix*, Fig. S3). Threading 10 sequence-diverse CIRs onto the PCHAS_1200500 structure generated models with a Phyre2 confidence score of 100%, supporting the conservation of this fold among PIRs from *P. chabaudi*. In contrast, threading 10 sequence-diverse RIFINs onto the PCHAS_1200500 structure gave an average confidence score of only 1% (range, 0.1 to 2.6%), as the RIFINs do not share the CIR protein fold.

To determine whether the fold is conserved in small VSAs from other rodent-infecting *Plasmodium* species, we selected proteins from *P. berghei* and *P. yoelii*. In each case, Phyre2 generated molecular models, based on threading these sequences onto the PCHAS_1200500 structure, with confidence values of 98.94 to 100%, strongly indicative of a conserved fold (Fig. 3*B* and *SI Appendix*, Fig. S3). To reveal the degree of conservation across the *P. berghei* and *P. yoelii* VSAs, we also generated sequence LOGOs for 135 *P*. *berghei* proteins and 1,011 *P. yoelii* proteins, allowing their comparison with sequence LOGOs for the CIRs. Plotting these onto the homology models showed that the *P. berghei* and *P. yoelii* VSAs share conserved sequence features with the CIRs, including conservation of two disulfide bonds, equivalent to conserved disulfide bonds C12-C165 and C42-C52 of the CIRs, as well as conservation of hydrophobic residues found in the domain core (Fig. 3*C* and *SI Appendix*, Fig. S4). These data show that it is highly likely that the *P. berghei* and *P. yoelii* VSAs adopt the same global structure as the *P. chabaudi* CIRs and are part of the same PIR protein family.

We next conducted the same analysis with VSAs from human-infecting malaria parasites, *P. vivax*, *Plasmodium malariae*, *Plasmodium ovale*, and *Plasmodium knowlesi*. Ten of each of these proteins were threaded onto the PCHAS_1200500 structure in Phyre2, and scores were obtained. In each case, the average score exceeded the 95% cutoff for a high-confidence model; 97.9% for *P. vivax*, 97.7% for *P. malariae*, 98.3% for *P. ovale*, and 98.7% for *P. knowlesi* (Fig. 3 and *SI Appendix*, Fig. S3). These findings indicate that small VSAs from these rodent-infecting and monkey-infecting clades are part of the PIR superfamily and that they share a common overall architecture.

We next studied small VSAs from *P. falciparum*. As described above, threading 10 diverse *P. falciparum* RIFIN sequences onto the CIR structure generated an average score of 1%, suggesting no structural similarity (Fig. 3*B*), agreeing with our comparison of the CIR structure with that of the variable domain of the RIFIN (Fig. 3*A*). However, no structure is available for the constant domain of a RIFIN. As this is smaller than the CIR ectodomain, we assessed whether the constant domain of the RIFIN shares structural homology with either the N-terminal or C-terminal lobes of the CIR. To do so, we threaded 10 sequence-diverse RIFIN constant domain sequences onto either the N-terminal (helices 1 to 5) or the C-terminal (helices 6 to 8) lobes of the CIR. In both cases, threading RIFIN sequences onto CIR structures gave confidence scores of <1%. A similar analysis of the *P. falciparum* STEVOR gave an average confidence score of 83.0% when we threaded the STEVOR sequences on the CIR structure and 24.7% when we threaded the STEVOR sequences onto the structure of the RIFIN variable domain. In neither case do these scores exceed the 95% threshold for high confidence. This compares with a confidence score of 94.3% when threading nine RIFIN structures onto the RIFIN variable domain structure (excluding one outlier).

Finally, we modeled the small VSAs from *Plasmodium reichenowi* and *Plasmodium gallinaceum*. *P. reichenowi*, like *P. falciparum*, is a member of the *Laverania* group of parasites. Here, threading 10 sequences onto the structure of the CIR generated an average score of 66.4%, while threading these sequences onto the RIFIN variable domain generated a score of 71.5%, both of which are below the confidence cutoff. These findings support the placement of the RIFINs and STEVORs of *P. falciparum* and the putative PIR proteins of *P. reichenowi* outside the PIR protein superfamily. Similarly, threading 10 sequences of small VSAs from bird-infective *P. gallinaceum* onto the CIR structure gave an average confidence score of 42.5%, while threading onto the RIFIN variable domain gave an average of 7.2%. This analysis suggests that the emergence of the PIR superfamily occurred after the separation of the rodent and simian-infective *Plasmodium* species from the *Laverania* and the bird-infective species, and indicates that the small VSAs found in the latter are not PIR proteins.

## Discussion

The structure of the ectodomain of a *P. chabaudi* CIR protein has allowed us to explore the fold which underlies this protein family and to determine the degree to which it has diversified. We find the CIRs to adopt a novel structure, consisting of eight α-helices, divided into two lobes. Despite their different architectures, the CIRs share properties with other proteins from the surfaces of *Plasmodium*-infected erythrocytes. Like the CIDR and DBL domains of *P. falciparum* PfEMP1 proteins, CIRs are built on a core α-helical scaffold. The most conserved residues are hydrophobic and aromatic or are disulfide bond-forming cysteine residues. These are all internal, stabilizing the structure. As disulfide bonds form only in the oxidizing extracellular environment, the presence of five such bonds in the structure of PCHAS_1200500, and the conservation of two of these across the rodent-infective malaria species, are indicative of a cell surface role for the PIRs.

Similar too, when comparing the CIR proteins with the PfEMP1, is that each has its own conserved structural fold, which is decorated with flexible surface loops. This has allowed extensive surface diversification in both sequence and loop length. This again is indicative of a surface location for the PIR proteins, with the development of antigenically distinct molecules to allow sequential deployment during an infection a potential driving force for diversification. In the CIRs, these domains are attached to the cell surface through a linker of variable length, perhaps acting as a protein–protein interaction module swinging on a flexible chain.

Families of small VSAs from across the *Plasmodium* genus have been proposed to be part of the PIR superfamily. However, the true extent of this family—and thus the origins, common or otherwise, of genes from different species—has remained unclear. As structures are more conserved than sequences, the structure of a PIR protein family member from *P. chabaudi* has allowed us to explore which other *Plasmodium* small VSAs are part of the PIR protein superfamily. This analysis predicts that the majority of other proteins examined, including those from the rodent-infective parasites *P. berghei* and *P. yoelii* and from human-infective species *P. ovale, P. vivax, P knowlesi*, and *P. malariae*, share the PIR protein fold and can be placed within the PIR superfamily. In contrast, analysis of RIFIN and STEVOR proteins of *P. falciparum* and the small VSAs of *P. reichenowi* and *P. gallinaceum* suggests that they do not share this fold, challenging their membership of the PIR superfamily. Examination of the *Plasmodium* family tree therefore suggests that the genesis of the PIR superfamily might have occurred after the divergence of the *Laverania* and bird-infective *Plasmodium* species from the rodent and simian-infective species, and reveals that the small VSAs from the *Plasmodium* species cannot be considered a homogeneous whole.

The placement of the RIFINs outside the PIR protein superfamily has functional consequences. The expression of different *P. chabaudi pir*s has recently been associated with acute or chronic *P. chabaudi* infection (28, 29). The growing evidence that RIFINs of *P. falciparum* bind to inhibitory immune receptors, such as LILRB1 and LAIR1 (17), and dampen immune signaling (33), is thus tantalizing. Could PIR proteins also interact with inhibitory immune receptors, thereby promoting chronic disease? It is still possible that they do, but the placement of the PIRs and the RIFINs in different protein families suggests that if this is indeed the case, it is the outcome of convergent evolution.

## Materials and Methods

### Protein Expression and Purification.

Synthetic genes encoding a panel of CIR ectodomains were cloned into a pTT3 vector, giving a C-terminal His6 tag. In the case of PCHAS_1200500, this contained five changes (S5A, T125A, S152A, S176A, and T179A) intended to remove putative *N*-linked glycosylation sites. These were transfected into HEK293F cells (Thermo Fisher Scientific) using polyethylenimine, and after 5 d harvested by centrifugation at 5,000 × *g*. The supernatant was buffer-exchanged into 20 mM Hepes pH 7.5, 150 mM NaCl, and 20 mM imidazole by tangential flow filtration, and the protein was purified by immobilized metal affinity chromatography using Ni^2+^-NTA resin, followed by size exclusion chromatography using a Superdex 75 10/300 column (GE Healthcare Life Sciences).

To methylate the protein, 20 mM dimethylamine-borane complex (ABC) and 40 mM formaldehyde were added, and the mixture was incubated for 2 h at room temperature. After incubation, this was repeated two more times, followed by the addition of 10 mM ABC and incubation of the reaction overnight. The protein was then purified by size exclusion chromatography as above.

### Crystallization, Data Collection, and Structure Determination.

For crystallization, methylated PCHAS_1200500 (2-264) was concentrated to 34 mg/mL, with trials carried out using vapor diffusion in sitting drops with a mixture of 100 nL of protein solution and 100 nL of well solution. Crystals were obtained after 40 d at 4 °C using a well solution of 0.1 M Hepes pH 6.5 and 45% wt/vol poly(acrylic acid sodium salt) 2100, and then cryocooled for data collection in liquid nitrogen.

Sulfur-SAD data were collected at the I23 long-wavelength beamline at the Diamond Light Source, using a wavelength of 2.75520 Å. Data were indexed and scaled using XDS, giving a resolution of 3 Å. Phases were obtained using SHELXD, and an initial model was then built using Buccaneer, with three molecules in the asymmetric unit of the crystal.

Native data were collected at the ID23-1 beamline at the European Synchrotron Radiation Facility and indexed and scaled using GrenADES fastproc, giving a resolution of 2.15 Å. The initial model from the Sulfur-SAD data was used as a search model, allowing it to be located within the higher-resolution dataset using Phaser MR. The structure was then built and refined using cycles of Coot and Buster.

### Circular Dichroism Analysis.

Circular dichroism experiments were conducted using a Jasco J815 CD spectrophotometer. The CIR was desalted into 20 mM sodium phosphate pH 7.5 and 150 mM NaF buffer using PD-10 columns (GE Healthcare) and diluted to a concentration of 0.26 mg/mL. A spectrum was obtained at 20 °C between 260 nm and 190 nm using a 1-mm path length, with measurements taken every 0.5 nm, subtracting a baseline determined using buffer. Ten equivalent spectra were averaged together.

### Sequence Analysis.

Small VSA sequences were extracted from *Plasmodium* genomes, including 198 amino acid sequences from the *P. chabaudi* AS v3 genome assembly (29), 135 sequences from the *P. berghei* ANKA genome assembly (25), 1,011 sequences from the *P. yoelii* 17X v3 genome assembly (41), 70 sequences from the *P. knowlesi* strain H genome assembly (42), 136 sequences from the *P. malariae* UG01 genome assembly (43), 1,495 sequences from the *P. ovale curtisi* GH01 genome assembly (43), 185 RIFIN sequences and 31 STEVOR sequences from the *P. falciparum* 3D7 genome assembly (44), 1,086 sequences from the *P. vivax* P01 genome assembly (8), 487 sequences from the *P. reichenowi* G01 genome assembly (45) and 20 sequences from the *P. gallinaceum* 8A genome assembly. All sequences were retrieved from PlasmoDB (46).

Sequences were aligned using MUSCLE v3.8.31 with default parameters. Sequence LOGOs were generated using http://weblogo.threeplusone.com. Homology models were produced using the Phyre2 server (40).

### Phylogenetic Analysis.

Evolutionary analyses were conducted in MEGA X (47, 48). The evolutionary history was inferred using the maximum likelihood method and the Whelan and Goldman frequency model (49). The tree with the highest log-likelihood value is shown. Initial trees for the heuristic search were obtained automatically by applying the Neighbor-Join and BioNJ algorithms to a matrix of pairwise distances estimated using a Jones–Taylor–Thornton model and then selecting the topology with the superior log-likelihood value. A discrete gamma distribution was used to model evolutionary rate differences among sites (five categories). The tree is drawn to scale, with branch lengths measured in the number of substitutions per site.

## Supplementary Material

Supplementary File

## Data Availability

Coordinates and structure factors have been deposited in the Protein Data Bank (PDB ID code 6ZYV). All other data and protein constructs are available from the authors on request.
