# Supplementary material for: Structure of the Plasmodium-interspersed repeat proteins of the malaria parasite
Source: Proc Natl Acad Sci U S A. 2020 Nov 30;117(50):32098–104. doi: 10.1073/pnas.2016775117 (PMC7749308; doi:10.1073/pnas.2016775117)
Supplement: Supplementary File [file pnas.2016775117.sapp.pdf]

Supplementary Information

Structure of the *Plasmodium*-interspersed repeat proteins of the malaria parasite

Thomas E. Harrison, Adam J. Reid, Deirdre Cunningham, Jean Langhorne and Matthew K. Higgins

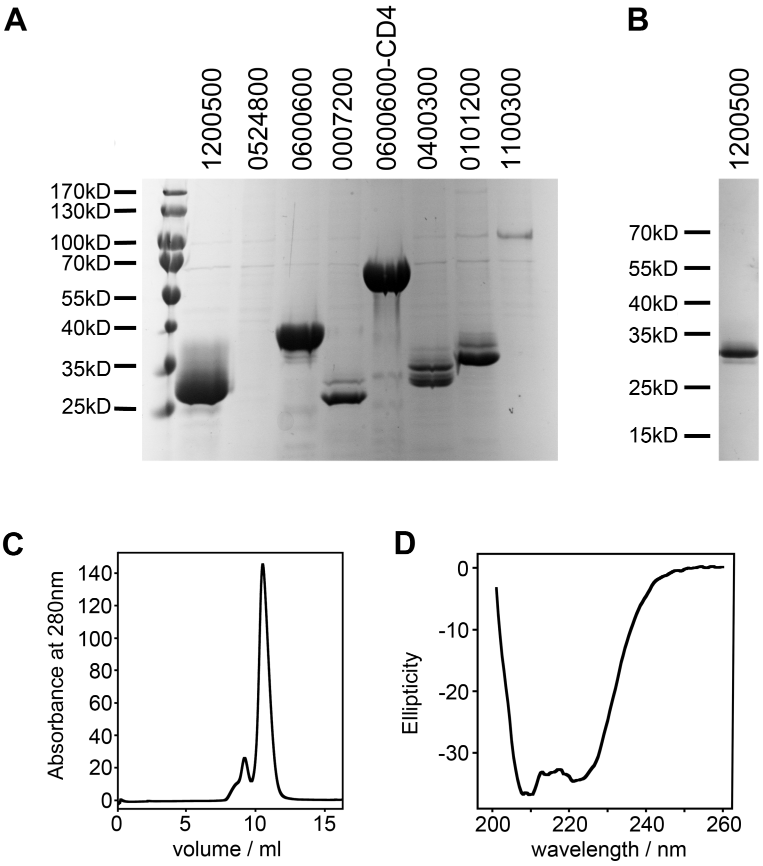

Supplementary Figure 1: Biophysical analysis of protein quality

A. Expression trial gel for CIR proteins expressed in HEK293 cells and purified using their His-tag before SDS-PAGE analysis. 06000600-CD4 also has a C-terminal CD4 tag. **B.** Coomassie-stained SDS PAGE gel; **C.** size exclusion column chromatogram (Superdex S75 10/30); and **D.** circular dichroism analysis of purified CIR 1200500.

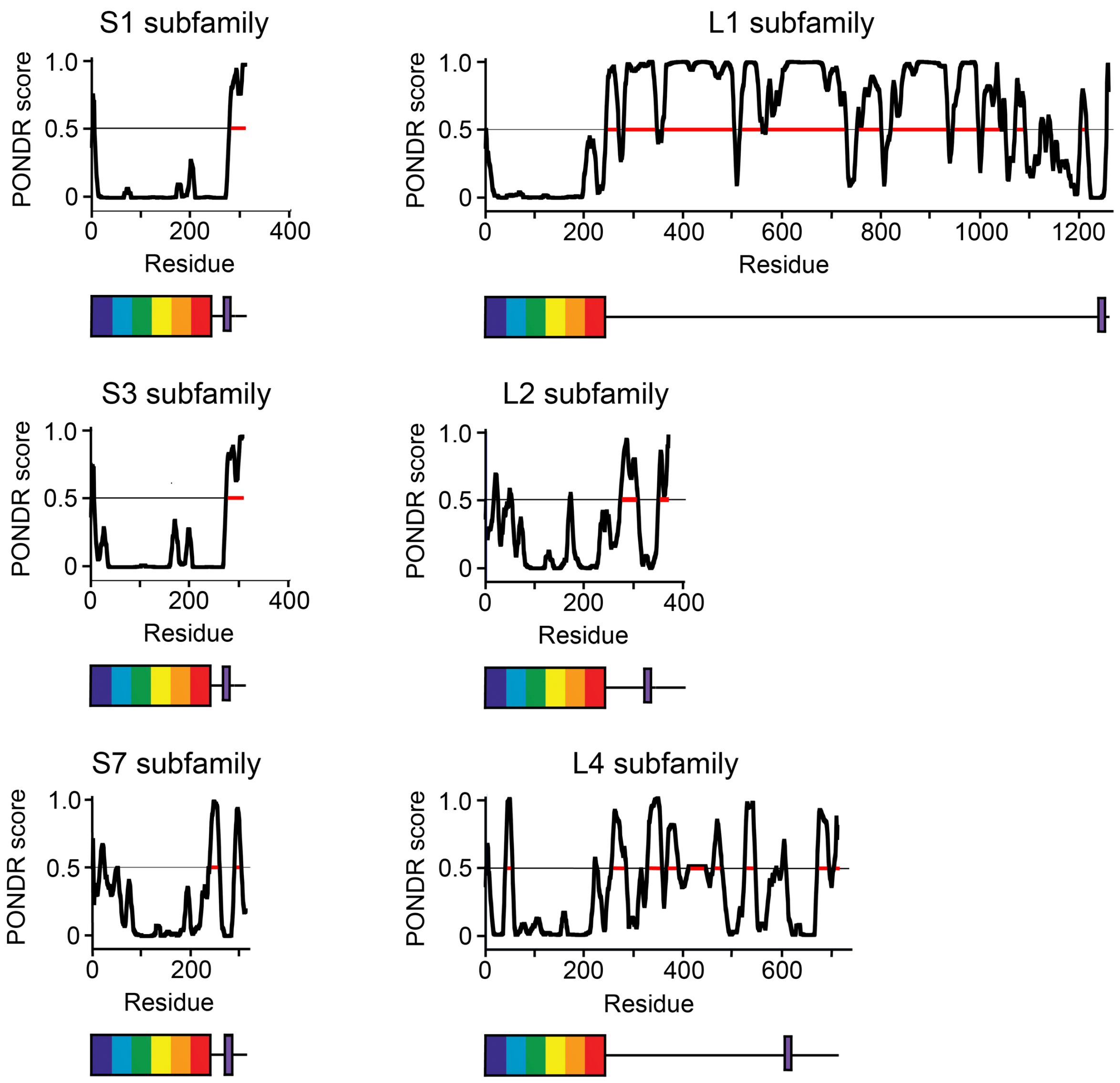

Supplementary Figure 2: Disorder prediction for different subfamilies of CIR protein.

Prediction of disorder for members of the six subfamilies of CIR proteins. In each case this is for the protein most close in sequence to the average sequence for that protein subfamily, as indicted by the sequence logo (for S1, PCHAS_0700300; for S3, PCHAS_1000300; for S7, PCHAS_0500200; for L1, PCHAS_0601000; for L2, PCHAS_0318500; and for L4, PCHAS_0301800). Prediction of disorder, determined using PONDR, is plotted against residue number. Below the plots are representations of the two proteins, showing the PIR protein domain as a rainbow box and the transmembrane helix in purple.

Supplementary Figure 3 – page 1

A

Family	Compared with	Confidence
P. chabaudi	CIR	100 (100-100)
P. berghei	CIR	99.9 (98.9-100)
P. yoelii	CIR	100 (100-100)
P. knowlesi	CIR	98.7 (98.2-98.9)
P. malariae	CIR	97.7 (88.9-99.0)
P. ovale	CIR	98.3 (94.1-99.6)
P. vivax	CIR	97.9 (91.6-99.8)
P. falciparum STEVOR	CIR	83.0 (65.0-91.0)
P. reichenowi	CIR	66.4 (0.4-88.8)
P. gallinaceum	CIR	42.5 (0-93.0)
P. falciparum RIFIN$_C$	CIR	1.1 (0.1-2.6)
P. falciparum RIFIN$_C$	CIR$_N$	0.4 (0.3-0.5)
P. falciparum RIFIN$_C$	CIR$_C$	0.3 (0.1-0.5)
P. falciparum STEVOR	RIFIN$_V$	24.7 (10.0-52.9)
P. reichenowi	RIFIN$_V$	71.5 (1.0-96.0)

B

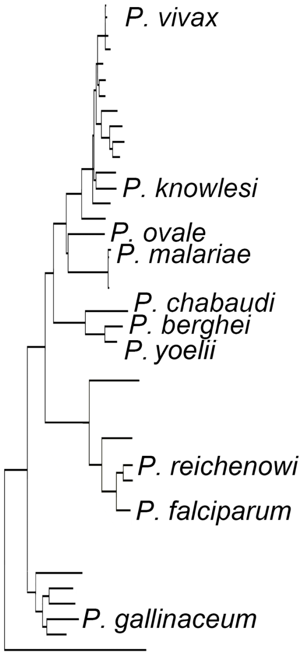

Supplementary Figure 3 – page 2

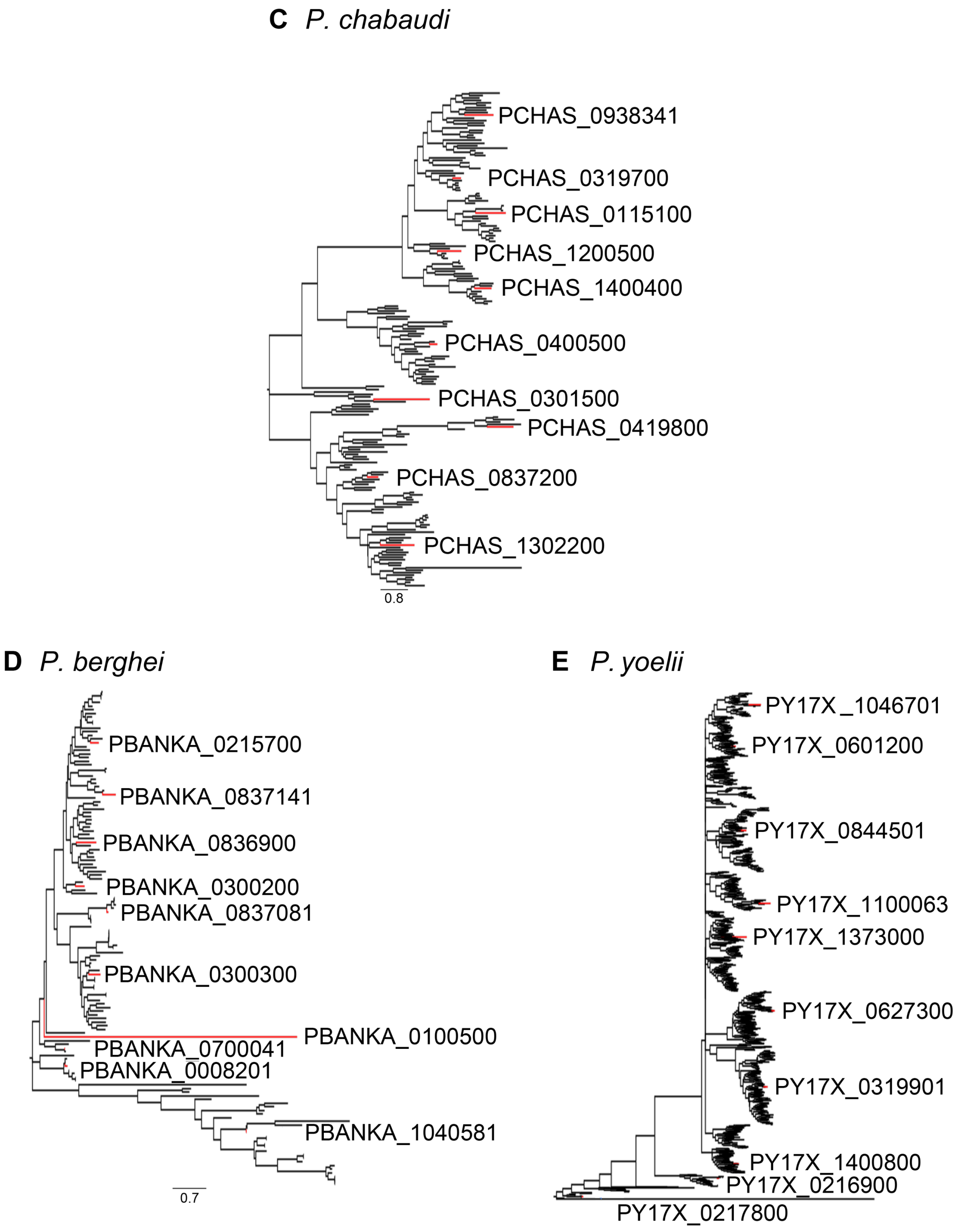

Supplementary Figure 3 – page 3

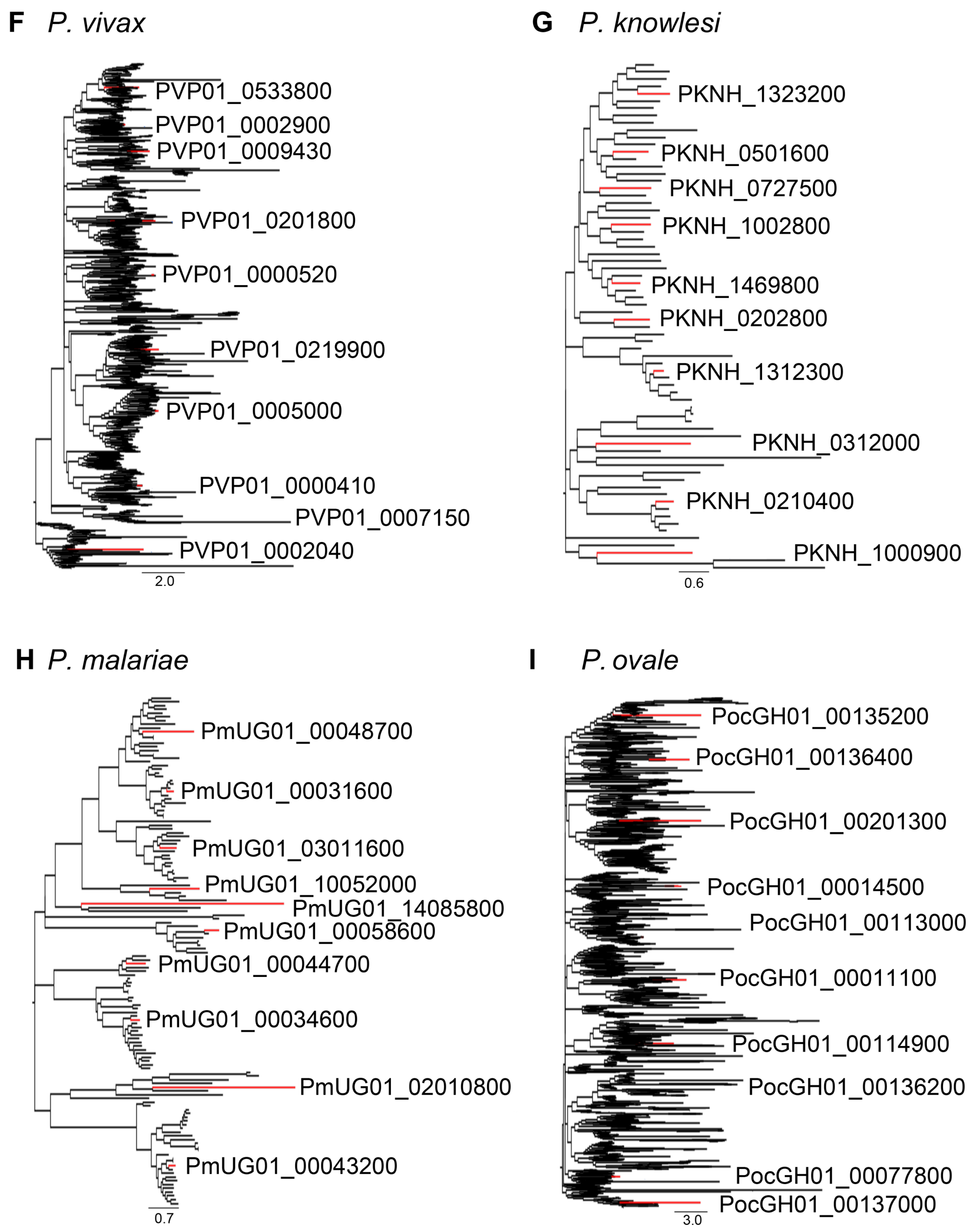

Supplementary Figure 3 – page 4

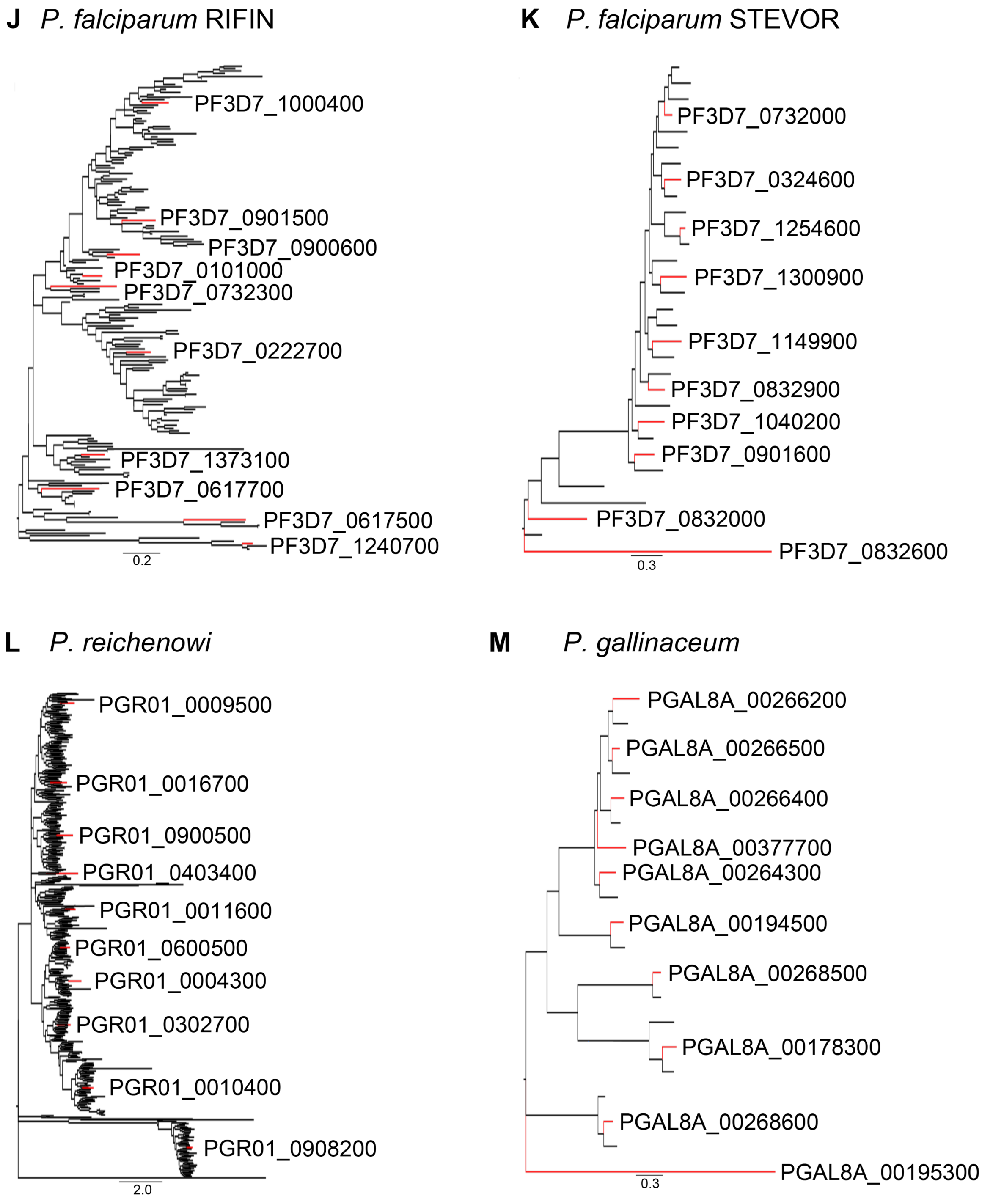

Supplementary Figure 3: Selection of small VSA proteins for analysis

A. The Phyre2 confidence scores for modelling of different small VSA proteins using the known structures of the CIR protein, its C-terminal domain (CIR_C: residues 152 – 241), its N-terminal domain (CIR_N: residues 4 - 151) or against the RIFIN variable domain ($RIFIN_V$). **B.** evolutionary tree for *Plasmodium* species, based on (50). **C.**-**M**. Evolutionary trees, based on sequence analysis of small VSA protein families from different *Plasmodium* species. The ten members of each family used for modelling studies are shown as red lines and are labelled.

Supplementary Figure 4 – page 1

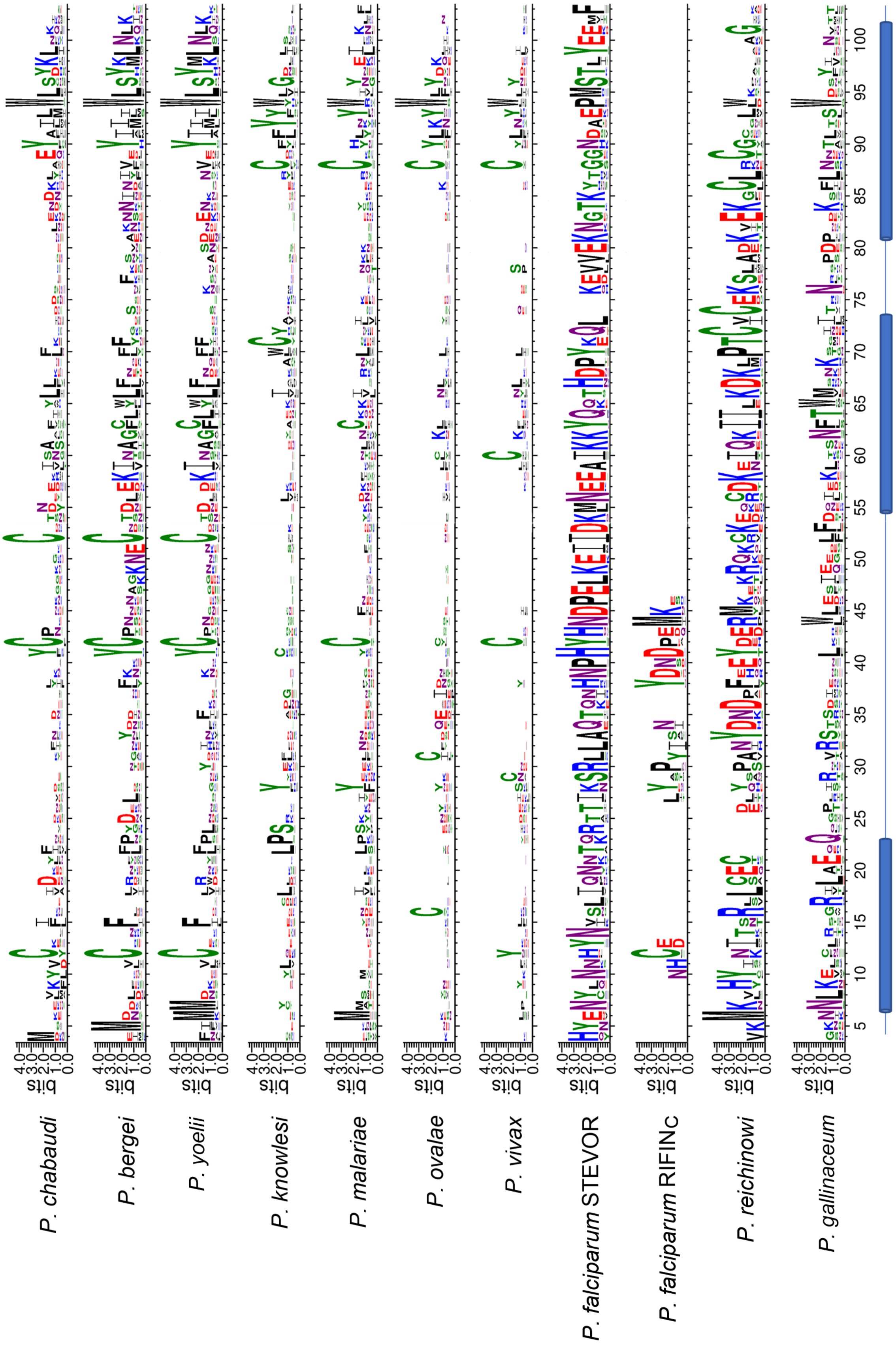

Supplementary Figure 4 – page 2

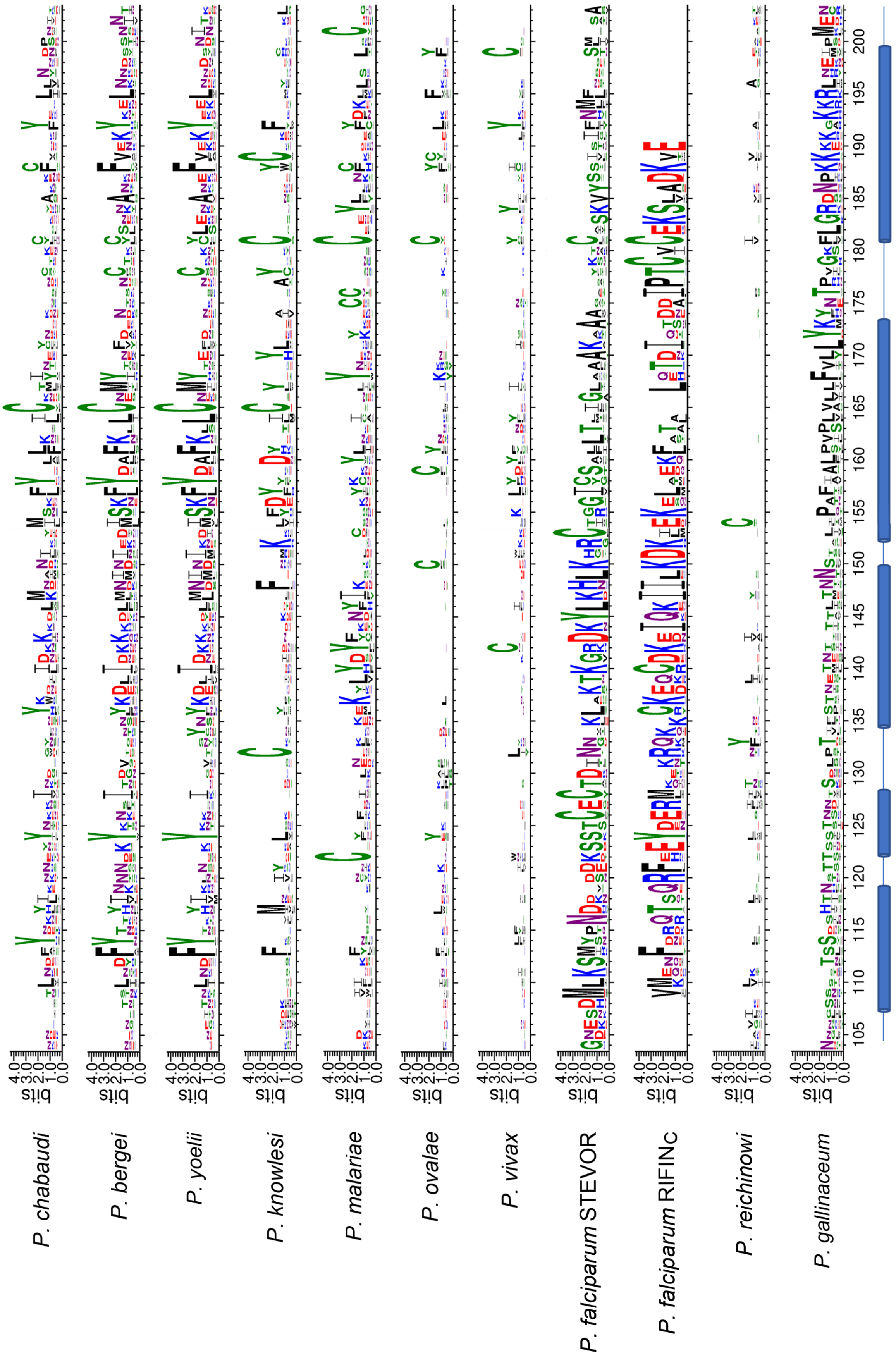

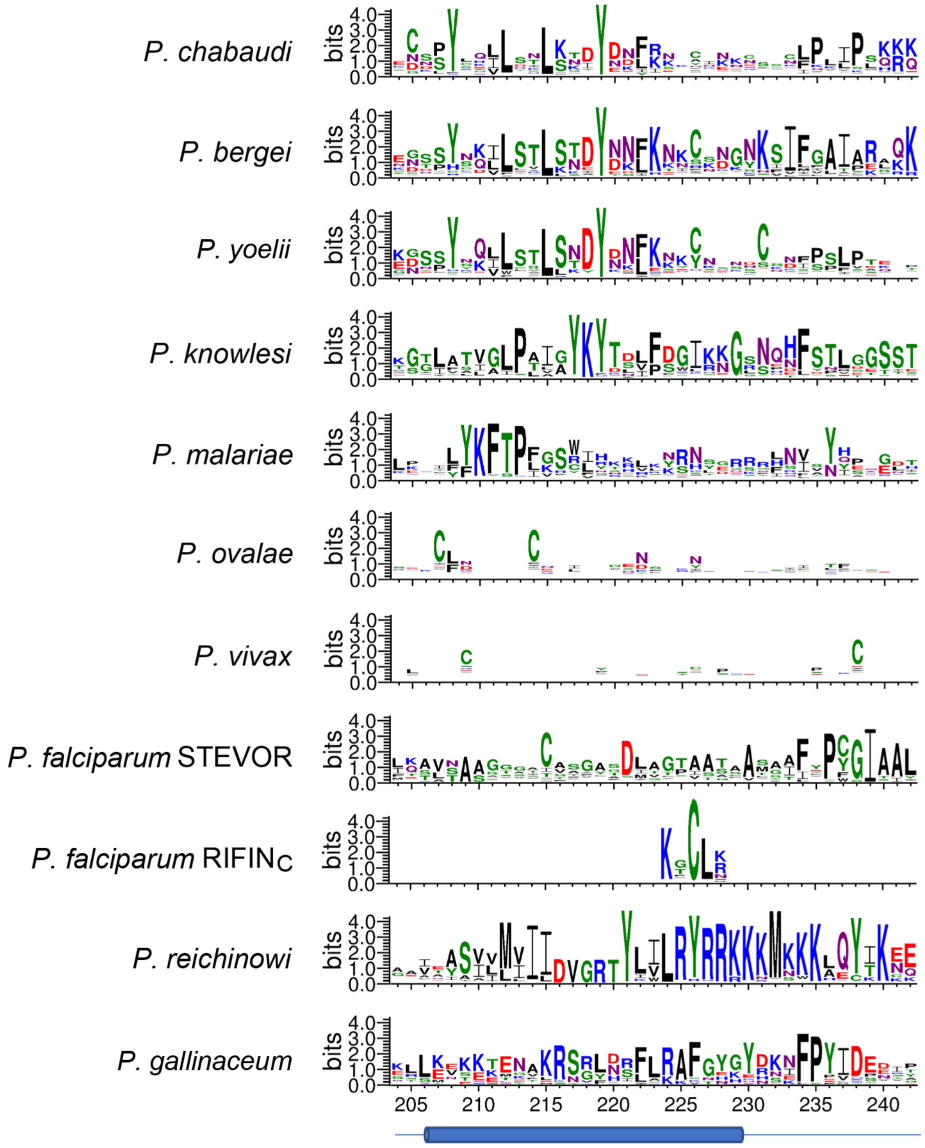

Supplementary Figure 4: conservation and diversity across the small VSAs

Sequences for small VSAs were aligned against the structure of PCHAS_1200500 and a sequence logo was generated, numbered according to PCHAS_1200500. The secondary structure of CIR PCHAS_1200500 is shown underneath the LOGO, with helices as cylinders and elements without secondary structure shown as a blue line. The sequences used were from *Plasmodium chabaudi* (198 sequences from the AS v3 genome assembly (29)), *Plasmodium berghei* (135 sequences from the ANKA genome assembly (25)), *Plasmodium yoelii* (1011 sequences from the 17X v3 genome assembly (41)), *Plasmodium knowlesi* (70 sequences from the strain H genome assembly (42)), *Plasmodium malariae* (136 sequences from the UG01 genome assembly (43)), *Plasmodium ovale curtisi* (1495 sequences sequences from the GH01 genome assembly (43)), *Plasmodium vivax* (1086 sequences from the *Plasmodium vivax* P01 (8)), *Plasmodium falciparum* (185 RIFIN sequences and 31 STEVOR sequences from the 3D7 genome assembly (44)), *Plasmodium reichinowi* 487 sequences from the G01 genome assembly (45), *Plasmodium gallinaceum* (20 sequences from the 8A assembly).

Table S1: Table of crystallographic statistics

**Native collection statistics**	
Space group	$P2_1\,2_1\,2_1$
Cell dimensions	
a, b, c (Å)	60.20, 63.58, 199.27
α, β, γ (°)	90, 90, 90
Wavelength	0.97166 Å
Resolution (Å)	49.84 – 2.15 (2.22 – 2.15)
Total Observations	79795 (6894)
Total Unique	42523 (3621)
R_{pim} (%)	4.0 (63.2)
$CC_{1/2}$	1.00 (0.55)
$I/\sigma(I)$	4.8 (1.3)
Completeness (%)	99.8 (99.9)
Multiplicity	1.9 (1.9)
Wilson B factor	54.4
**Sulphur-SAD Collection Statistics**	
Space group	$P2_1\,2_1\,2_1$
Cell dimensions	
a, b, c (Å)	63.81, 65.61, 201.95
α, β, γ (°)	90, 90, 90
Wavelength	2.755200
Resolution (Å)	62.41 – 3.00 (3.20-3.00)
Total Observations	387923 (57599)
Total Unique	31506 (5127)
R_{pim} (%)	6.2 (114.3)
$CC_{1/2}$	1.00 (0.74)
$I/\sigma(I)$	22.51 (1.82)
Completeness (%)	96.0 (88.9)
Multiplicity	12.3 (11.2)
CC_{anom}	0.62 (0.02)
Anomalous Signal ($\lvert F(+)-F(-)\rvert/\sigma$)	1.54 (0.70)
**Refinement Statistics**	
Reflections	42587
R_{work} / R_{free} (%)	21.2/23.6
Average B factor	64.0
Number of residues	700
R.m.s deviations	
Bond lengths (Å)	0.008
Bond angles (°)	0.93
Ramachandran plot	
Favored (%)	96.2
Allowed (%)	3.8
Outliers (%)	0.0
